# Identification and validation of a prognostic risk-scoring model based on the level of *TIM-3* expression in acute myeloid leukemia

**DOI:** 10.1038/s41598-023-42700-2

**Published:** 2023-09-20

**Authors:** Wanxue Huang, Shasha Zheng, Qi Wang, Na Zhao, Zhiguo Long

**Affiliations:** 1 Department of Hematology, Fudan University Affiliated Pudong Medical Center, Shanghai, China; 2 Jiangsu Province Hospital of Chinese Medicine, Affiliated Hospital of Nanjing University of Chinese Medicine, Nanjing, China

**Keywords:** Cancer genetics, Cancer stem cells, Tumour biomarkers, Tumour immunology

## Abstract

Acute myeloid leukemia (AML) is characterized by an unfavorable prognosis due to the presence of self-renewing leukemic stem cells (LSCs). The presence of T-cell immunoglobulin mucin-3 (*TIM-3*) on the surface of LSCs has been observed in various types of human AML, exerting an impact on the prognostic outcome. Exploring the hub genes associated with varying levels of *TIM-3* expression offers a valuable approach to enhance our understanding of the underlying mechanisms involving *TIM-3* and to identify potential prognostic indicators in AML. Nevertheless, to date, no research studies have reported a prognostic model that relies on the level of *TIM-3* expression. In our study, we screen the hub-genes based on different expression level of *TIM-3* through WGCNA. The prognostic risk-scoring model was constructed based on hub-genes. The results show the risk prognostic model has extraordinary ability to predict prognosis in both the training and validation sets. The high-risk group present poor prognosis with mutation of *NPM1*, *TP53* (Multiple Hit) and *FLT3*(multiple hit), while *IDH2* (Missense Mutation), *MUC16* (Multiple Hit/Missense Mutation) occur mutation in low-risk group presenting favorite prognosis than high-risk group. Leukocyte cell–cell adhesion, regulation of T cell activation and I-κB kinase/NF-κB signaling enriched in high-risk group, involving in HSCs or LSCs anchoring to BM, which implicated in LSCs survival and chemotherapy resistance. *B7-H3* (*CD276*) and *CD276* would be the potential immune targets in high-risk group. The risk score model may help in distinguishing immune and molecular characteristics, predicting patient outcomes.

## Introduction

Acute myeloid leukemia (AML) is a disease characterized by an unfavorable prognosis, originating from self-renewing leukemic stem cells (LSCs)^1^. A major contributing element to AML's incurable nature is resistance to treatment^2^. LSCs endure in individuals suffering from AML, exhibiting resilience against traditional therapeutic methods. These LSCs display varied clones and function as reservoirs, thereby contributing to the recurrence, relapse, or intensification of the ailment into more aggressive phenotypes^3^. Throughout a relapse, there exists a proliferation of the LSC populace^4^. Leukemia development is facilitated by reciprocal interactions between leukemic cells and the surrounding microenvironment^5^. Enacting a treatment approach centered on aiming at LSCs harbors potential for diminishing the likelihood of AML reappearance and potentially enhancing the overall recovery rate.

T-cell immunoglobulin mucin domain-containing protein 3 (*Tim-3*) operates as a inhibitor of inherent and adaptable immune reactions^6–8^. *TIM-3* expression has been observed on the surface of LSCs in various types of human AML, whereas hematopoietic stem cells (HSCs) lack expression^9,10^. A recent study revealed that *Tim-3* protein is functionally expressed by CD34 + CD38—LSCs in AML, while it remains absent in healthy HSCs, as well as in myeloerythroid and lymphoid progenitor populations^10^. Furthermore, by analyzing somatic mutations, it has been determined that multistep leukemogenesis arises from self-renewing HSCs, highlighting *Tim-3* as a potential target for selectively eliminating LSCs while preserving residual HSCs^11^. In a recent investigation involving 302 AML patients, *Tim-3* was detected in LSCs at the time of initial diagnosis in 78.5% of cases^12,13^. Moreover, *Tim-3* serves as an immune checkpoint and plays a critical role in immune responses during AML^14^. Therefore, *Tim-3* holds promise as a potential therapeutic marker in AML.

**Figure 1:**
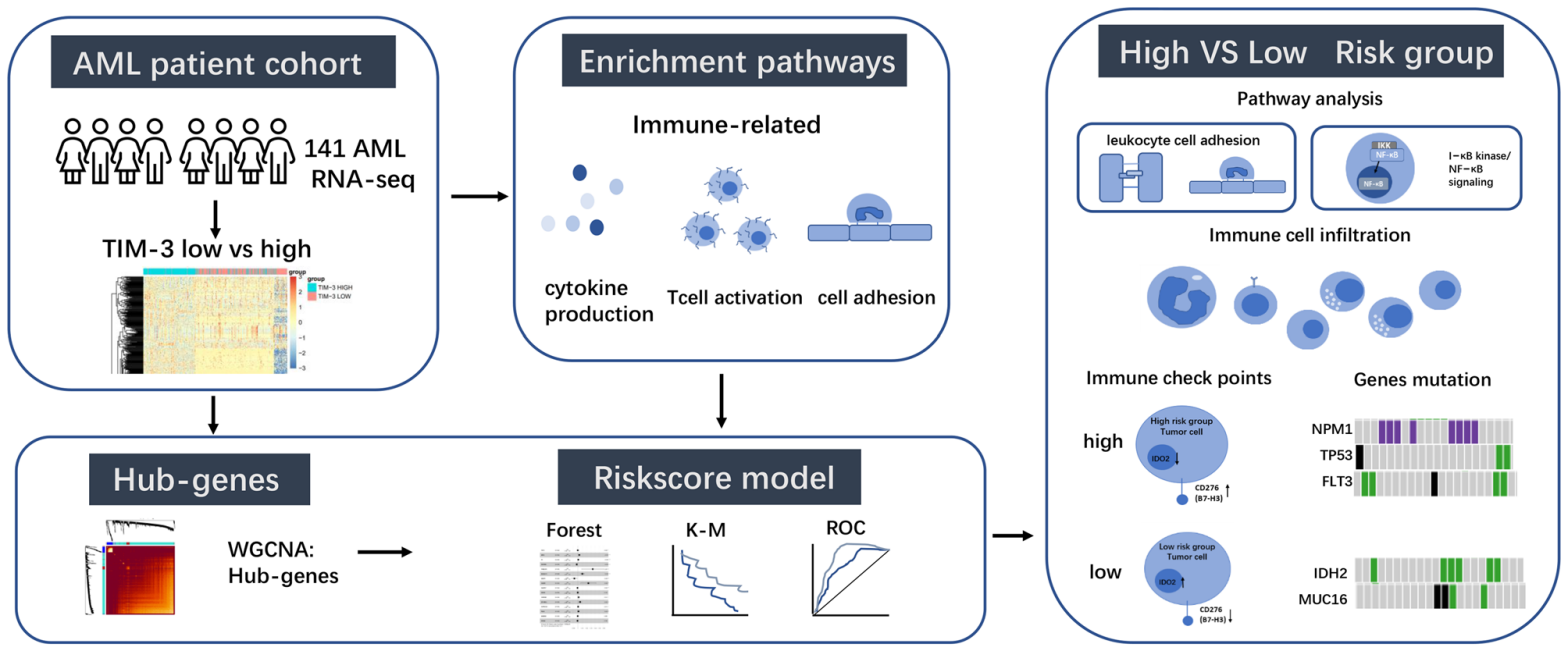
Flowchart of this article.

Though a study reveals the expression of *Tim-3* may affect prognosis in AML patients^13^. The precise mechanisms through which *TIM-3* impacts the prognosis of individuals with AML are currently lacking in comprehensive understanding. The therapeutic potential and underlying mechanisms of *Tim-3* in AML necessitate further investigation. Despite extensive research aimed at identifying prognostic markers in AML, a validated biomarker capable of accurately predicting response to immunotherapy and overall survival (OS) has yet to be identified. This emphasizes the imperative of discovering a prognostic biomarker specifically for immunotherapy in AML^15^. Investigating the hub genes associated with varying levels of *TIM-3* expression presents an opportunity to gain insights into the underlying mechanisms of *TIM-3* and identify prognostic markers in AML. However, there is no prognostic model based on level of *TIM-3* expression. In our study, we employed weighted gene co-expression network analysis (WGCNA) to identify hub genes in the high and low *TIM-3* expression groups. Subsequently, we developed a risk prognostic model based on the identified hub genes. The risk prognostic model has extraordinary ability to predict prognosis in both the training and validation sets, closing to European Leukemia Net (ELN) recommendations in decision curve analysis (DCA). The high-risk group exhibits a poor prognosis associated with mutations in *NPM1*, *TP53* (Multiple Hit), and *FLT3* (Multiple Hit), whereas the low-risk group is characterized by mutations in *IDH2* (Missense Mutation) and *MUC16* (Multiple Hit / Missense Mutation). Leukocyte cell–cell adhesion, regulation of T cell activation, and I-κB kinase/NF-κB signaling are enriched in the high-risk group, and these processes are involved in the anchoring of HSCs or LSCs to the bone marrow(BM), implicating their role in LSC survival and chemotherapy resistance. Additionally, *B7-H3* (*CD276*) and *IDO2* may serve as potential immune targets in the high-risk group. The risk score model demonstrated superior predictive power in AML with high *TIM-3* expression. The prognostic models built upon these hub genes have the potential to offer enhanced predictive insights (Fig. 1).

## Results

### DEGs of *TIM-3* expression

**Figure 2:**
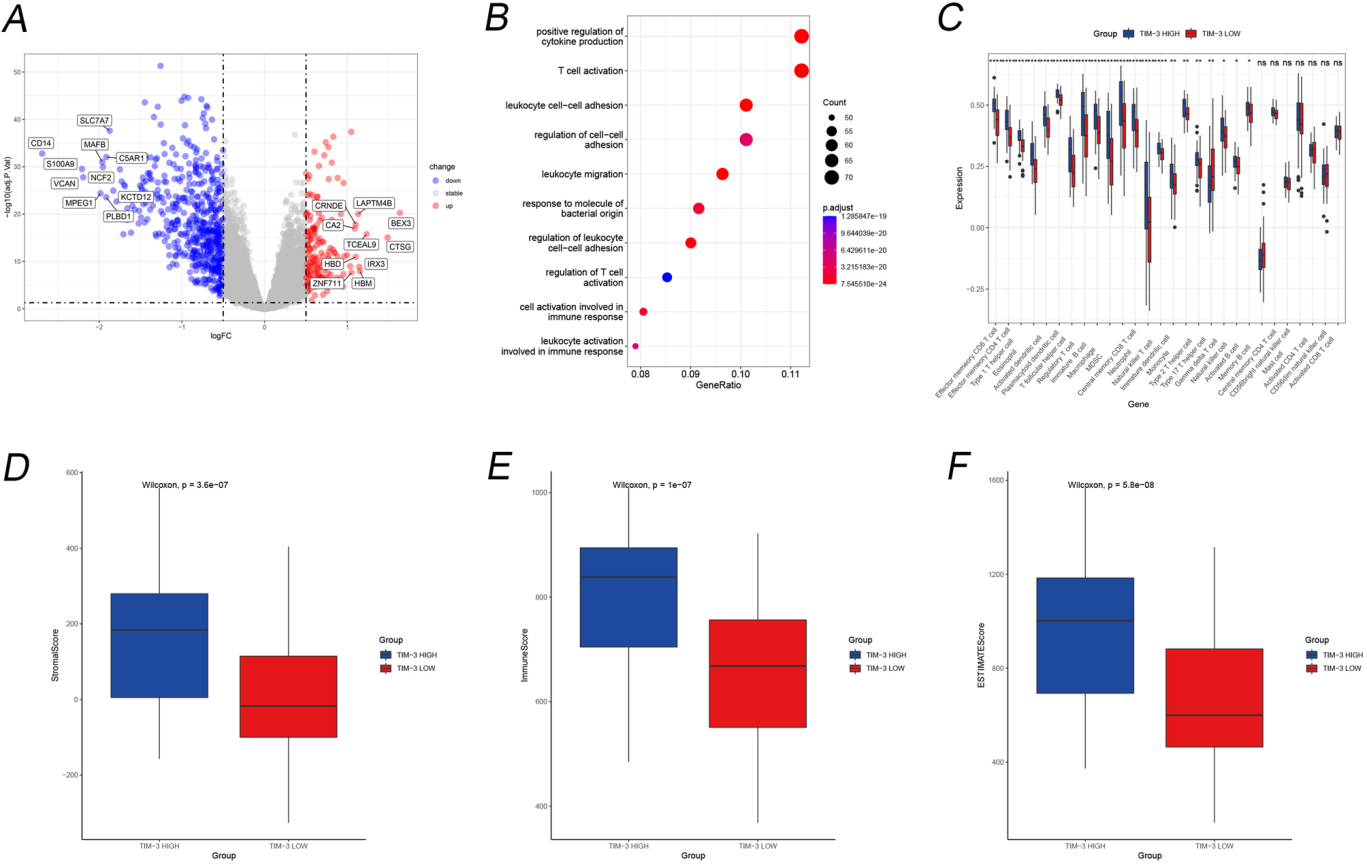
Exploration based on the high and low expression of *TIM-3*. (**A**) The Volcano plot pf DEGs based on expression of *TIM-3*. The top 10 genes for up- and down-regulation are labeled separately. (**B**) The pathway of DEGs. (**C**) Immune cells infiltration between high vs low group in expression of *TIM-3*. (**D**) StromalScrore in high versus low group based on *TIM-3* expression. (**E**) ImmuneScore in high versus low group based on TIM—Fig. 3 expression (**F**) ESTIMATEScore in high versus low group based on *TIM-3* expression.

In the analysis of differential gene expression between high and low *TIM-3* expression groups, a total of 7652 genes showed significant differential expression (Fig. 2A). Supplementary Fig. S1A provides the corrected data for the top 20 DEGs. Following the analysis of enrichment pathways for the DEGs (Fig. 2B), we identified the top three pathways: positive regulation of cytokine production, T cell activation, and leukocyte cell–cell adhesion. Building upon these discoveries, we subsequently examined the immune cells and scores of the immune microenvironment in the two groups exhibiting high and low *TIM-3* expression. We detected disparities in the manifestation of diverse T cells between the groups displaying high and low *TIM-3* expression (Fig. 2C). Additionally, the immune scores varied among the distinct groups, with increased estimateScores, StromalScore, and immuneScores discerned in the group exhibiting high *TIM-3* expression. Remarkably, immune-associated genes held a crucial position among the DEGs linked to *TIM-3* manifestation (Fig. 2D–F).

### Immune-related hub genes

**Figure 3:**
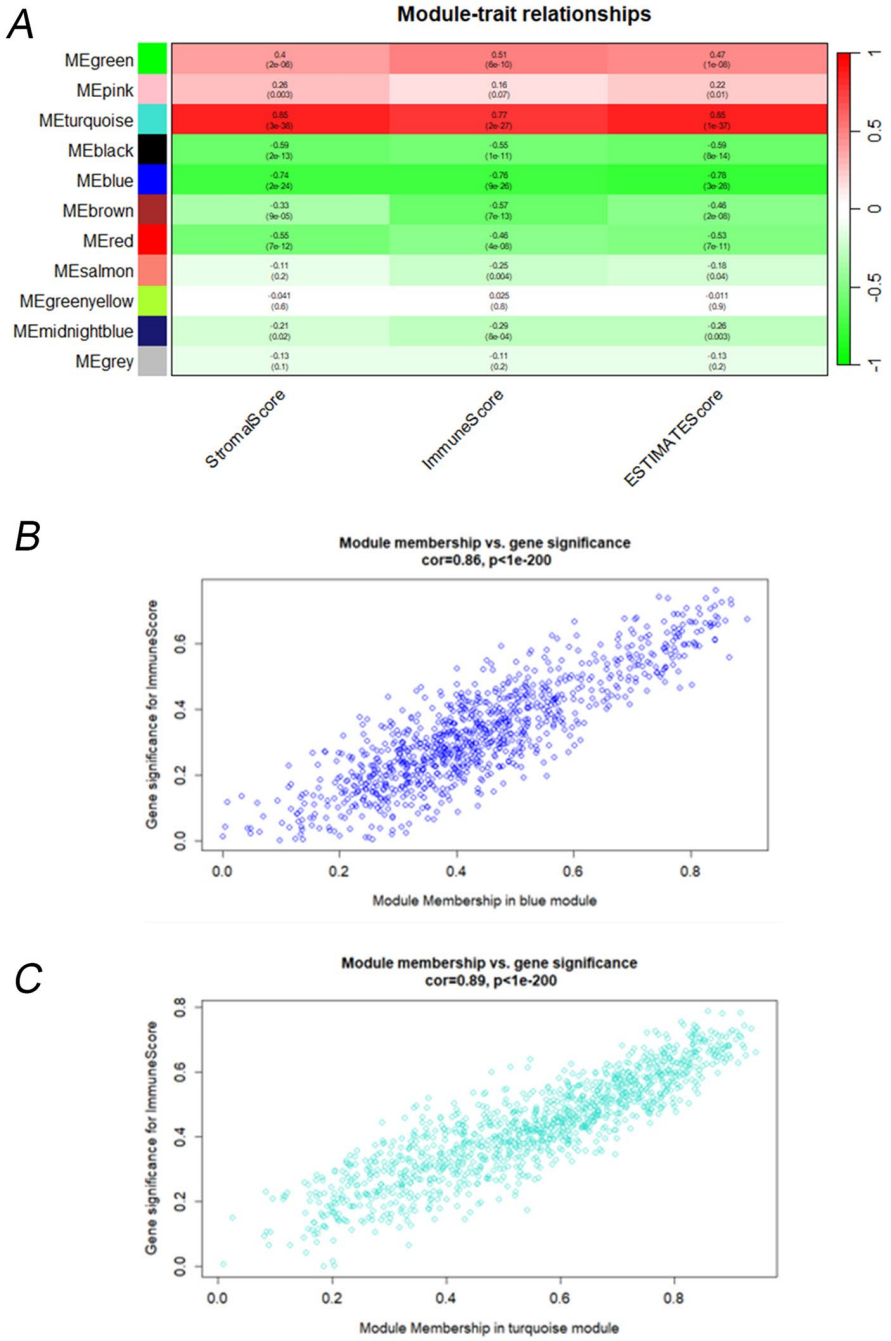
Immune-related hub genes based on DEGs of expression of *TIM-3* through WGCNA. (**A**) Module trait relationship. (**B**) The correlation between module membership in blue module and ImmuneScore. (**C**) The correlation between module membership in turquoise module and ImmuneScore.

To gain comprehensive insights into immune-related hub genes, we performed WGCNA on the set of candidate genes. By employing WGCNA, we aimed to elucidate the intricate relationships and co-expression patterns among these genes, thereby facilitating a deeper understanding of their involvement in immune processes. The ideal soft-thresholding exponent was established as 5 (Supplementary Fig. S1 B–C). Subsequently, we detected a cumulative of 11 modules (Fig. 3A). By assessing the Pearson correlation coefficient between each module and the sample feature, we noticed that the blue and turquoise module demonstrated a robust correlation with immune scores. Consequently, we chose the genes within two modules as hub genes for subsequent analysis (Fig. 3B,C).

### Construction of risk score model

**Figure 4:**
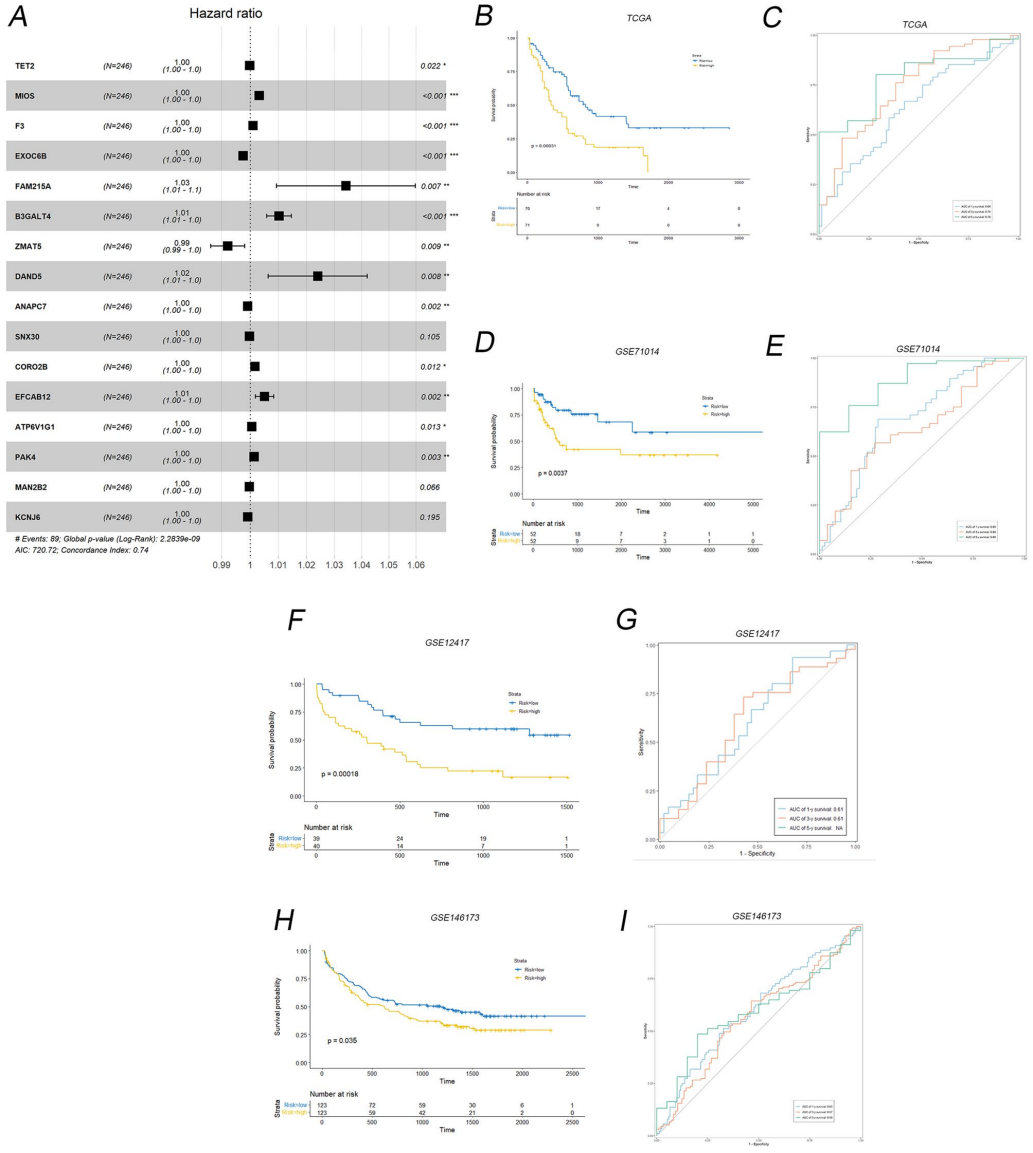
Construction and validation of risk score model. (**A**) 22 genes in immune-related hub genes to form the risk score model. (**B**) Kaplan Meier (K–M) plot of TCGA based on high versus low risk group. (**C**) Receiver operating characteristic (ROC) curves of TCGA. (**D**) K–M plot of GSE71014 based on high versus low risk group. (**E**) ROC curves of GSE71014. (**F**) K–M plot of GSE12417 based on high versus low risk group. (**G**) ROC curves of GSE12417. (**H**) K–M plot of GSE146173 based on high versus low risk group. (**I**) ROC curves of GSE146173.

To identify independent prognostic genes, we conducted multivariate survival analysis (Kaplan–Meier, *p* < 0.05) on immune-related hub genes, specifically examining overall survival (OS). Among the hub genes, we identified significant OS influencers, which were utilized to develop a model through multivariate Cox regression analysis. Subsequently, we selected 22 genes from the immune-related hub genes to create the risk score model (Fig. 4A). This model served as a prognostic index for all cancer samples, computed using a formula (Supplementary Table 1). By employing the median risk score as the cut-off, patients with a low-risk score displayed superior overall survival compared to those with a high-risk score (*P* = 0.00031, Fig. 4B). Additionally, to fully validate the reliability of the model, we chose different databases: GSE71014 (Fig. 4D,E), GSE12417(Fig. 4F,G), GSE146173(Fig. 4H,I) as external validation. This included samples of cytogenetically normal AML (CN-AML) and secondary AML, treatment-resistant AML, with a view to approximating real-world. Summary of patients’ clinical characteristics from 4 datasets was shown in Table1. The results exhibited consistent results with the TCGA dataset, confirming that patients with a low-risk score experienced significantly better prognosis than those with a high-risk score. Moreover, the receiver operating characteristic (ROC) curves in both the training and validation sets demonstrated the robust predictive prognostic capability of the risk score model at 1, 3, and 5-year survival durations, particularly at the 5-year mark (Fig. 4C,E).

**Table 1:** Summary of patients’ clinical characteristics from 4b datasets.

Patient’s parameters	TCGA	GSE71014	GSE146173	GSE12417
Age, years/n(%)				
< 65	98(69.5)	–	172(69.9)	47(59.5)
≥ 65	43(30.5)	–	74(30.1)	32(40.5)
WBC, × 10^9^/L/n(%)				
< 10	53(37.6)	–	103(41.9)	–
≥ 10	87(61.7)	–	143(58.1)	–
NA	1(0.7)	–	–	
PB blasts, %/n(%)				
< 40	16(11.3)	–	–	–
≥ 40	125(88.7)	–	–	–
BM blasts, %/n(%)				
< 50	88(62.4)	–	–	79(100)
≥ 50	53(37.6)	–	–	–
Risk/n(%)				
Favorable	30(21.3)	–	65(26.4)	–
Intermediate/normal	77(54.6)	–	127(51.6)	–
Poor	32(22.7)	–	46(18.7)	–
NA	2(1.4)	–	8(3.2)	–
OS/n(%)				
Alive	52(36.9)	68(65.4)	97(39.4)	33(41.8)
Dead	89(63.1)	36(34.6)	149(60.6)	46(58.2)
Survival rates, year/(%)				
1	56	52.9	62.2	59.5
3	18.4	24	41.1	26.6
5	5	14.4	8.1	–
AUC, year/(%)				
1	0.64	0.69	0.60	0.61
3	0.74	0.64	0.57	0.61
5	0.78	0.89	0.59	–
Platform	–	GPL10558	IlluminaHiSeq 1500	GPL570
Total	141	104	246	79

Correlation analysis of clinical parameters results showed that the risk score was correlated with patients’ Gene mutation (*FLT3*, *IDH1*, *NPM1c*), no significant correlation was found between risk score and other clinical parameters (Table 2). Multivariate survival analysis found that risk prognostic model was the independent prognostic factor for AML patients (Table 3).

**Table 2:** Comparison of TCGA patients’ clinical characteristics in two groups.

Patient’s parameters	Total	Low (n = 70)	High (n = 71)		P*
Age, years/n(%)					0.7565
< 65	98(69.5)	50(61.4)	48(46.5)		
≥ 65	43(30.5)	20(38.6)	23(53.5)		
WBC, × 10^9^/L/n(%)					0.7275
< 10	53(37.6)	28(40)	25(35.2)		
≥ 10	87(61.7)	42(60)	45(63.4)		
NA	1(0.7)	–	1(1.4)		
PB blasts, %/n(%)					0.4084
< 40	16(11.3)	10(14.3)	6(8.5)		
≥ 40	125(88.7)	60(85.7)	65(91.5)		
BM blasts, %/n(%)					0.3281
< 50	88(62.4)	47(67.1)	41(57.7)		
≥ 50	53(37.6)	23(32.9)	30(42.3)		
Risk/n(%)					0.1917
Favorable	30(21.3)	19(26.8)	11(15.5)		
Intermediate/normal	77(54.6)	34(48.6)	43(60.6)		
Poor	32(22.7)	17(24.3)	15(21.1)		
NA	2(1.4)	–	2(2.8)		
FAB classifications/n(%)					0.7437
M0	14(9.9)	8(11.4)	6(8.5)		
M1	32(22.7)	17(24.3)	15(21.1)		
M2	34(24.1)	14(20)	20(28.2)		
M3	14(9.9)	8(11.4)	6(8.5)		
M4	28(19.9)	15(21.4)	13(18.3)		
M5	15(10.7)	5(7.1)	10(14)		
M6	2(1.4)	1(1.4)	1(1.4)		
M7	1(0.7)	1(1.4)	–		
NA	1(0.7)	1(1.4)	–		
Gene mutation					
FLT3	41(29)	14(20)	27(38)		0.01851
IDH1	26(18.4)	18(25.7)	8(11.3)		0.0461
NPM1c	33(23.4)	10(14.3)	23(32.4)		0.01927
OS/n(%)					< 0.001
Alive	52(36.9)	36(51.4)	16(22.5)		
Dead	89(63.1)	34(48.6)	55(77.5)		

WBC, white blood cell; BM, bone marrow; PB, peripheral blood; NA, not applicable.

*Chi-aquare test.

**Table 3:** Univariate and multivariate overall survival analysis in the TCGA.

Variable	Univariate analysis			Multivariate analysis		
	Coef	HR(95%CI)	*P*	Coef	HR(95%CI)	*P*
Risk model	2.7	1.9–3.9	< 0.001	3.2	1.8–5.5	< 0.001
Age(≥ 65 vs. < 65)	2.9	1.9–4.5	< 0.001	2.4	1.5–3.7	< 0.001
WBC(≥ 10 × 10^9^/L vs < 10)	4.6	1.1–19	0.033	2.7	0.7–11.2	0.16
PB blasts(≥ 40 vs. < 40)	1.3	0.68–2.4	0.44	–	–	–
BM blasts(≥ 50 vs. < 50)	1.3	0.86–2	0.21	–	–	–
ELN risk stratification	1.8	1.3–2.5	< 0.001	1.696	1.2–2.4	0.004
Gene mutation						
FLT3 (mutated vs. wild)	1.3	0.86–2.1	0.19	–	–	–
IDH1 (mutated vs. wild)	0.72	0.31–1.7	0.44	–	–	–
NPM1c (mutated vs. wild)	1.1	0.22	0.64	–	–	–

WBC, white blood cell; BM, bone marrow; PB, peripheral blood.

### Molecular characteristics of different risk score model

**Figure 5:**
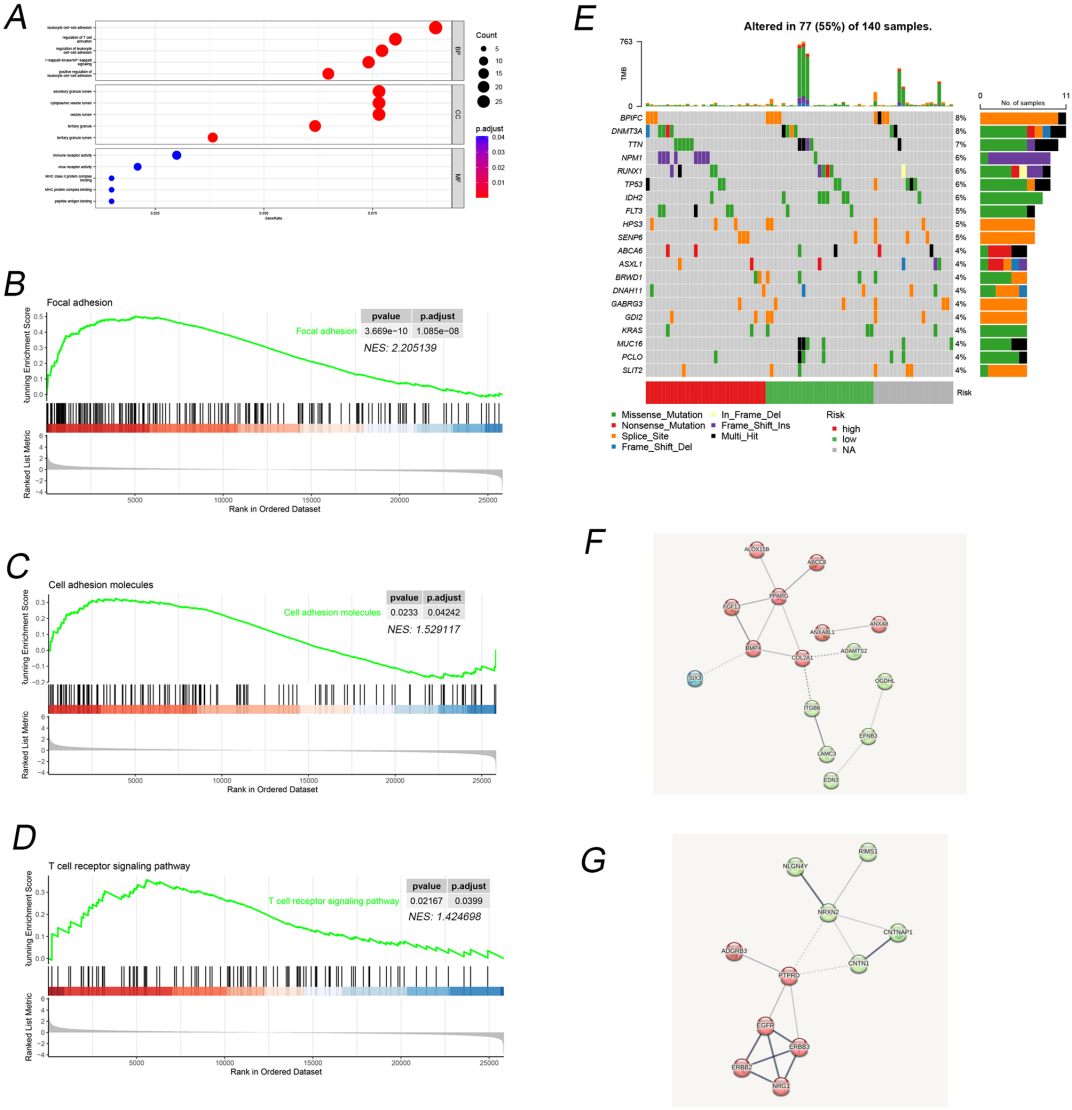
Molecular characteristics of different risk score model. (**A**) The pathway of DEGs based on high vs low risk group. (**B**) Gene set enrichment analysis (GSEA) of focal adhesion. (**C**) GSEA of cell adhesion molecules. (**D**) GSEA of T cell receptor signaling pathway. (**E**) Oncoplot based on different risk groups. (**F**) PPI network of up genes in DEGs’ high versus low risk group. (**G**) PPI network of down genes in DEGs high versus low risk group. Each node represents each protein. The thickness of the line represents the strength of the association between the proteins.

GO, GSEA was performed to determine the gene sets enriched in different risk groups. The DEGs of different risk groups enrich in leukocyte cell–cell adhesion, regulation of T cell activation and I-κB kinase/NF-κB signaling (Fig. 5A). The results of GSEA are consistent with the result of functional enrichment analysis (Fig. 5B–D). Next, we analyzed gene mutations to gain further biological insight into the risk score groups. We found *NPM1* (Frameshift insertions), *TP53* (Multiple Hit) present with high-risk group, while *IDH2* (Missense Mutation), *MUC16* (Multiple Hit/Missense Mutation) in low-risk group (Fig. 5E). We also found that *DNMT3A* occurs frameshift insertions and nonsense mutation in the high-risk group, while multiple hit and splice site in the low-risk group; *RUNX1* occurs multiple hit in the high-risk group, while nonsense mutation in the low-risk group; *FLT3* occurs multiple hit in the high-risk group and *DNAH11* occurs (Frameshift Deletion) in the low-risk group. The Protein–Protein Interaction(PPI) Networks analysis for DEGs (high vs low risk groups) were shown in Fig. 5F,G.

### Immune characteristics of different risk score model

**Figure 6:**
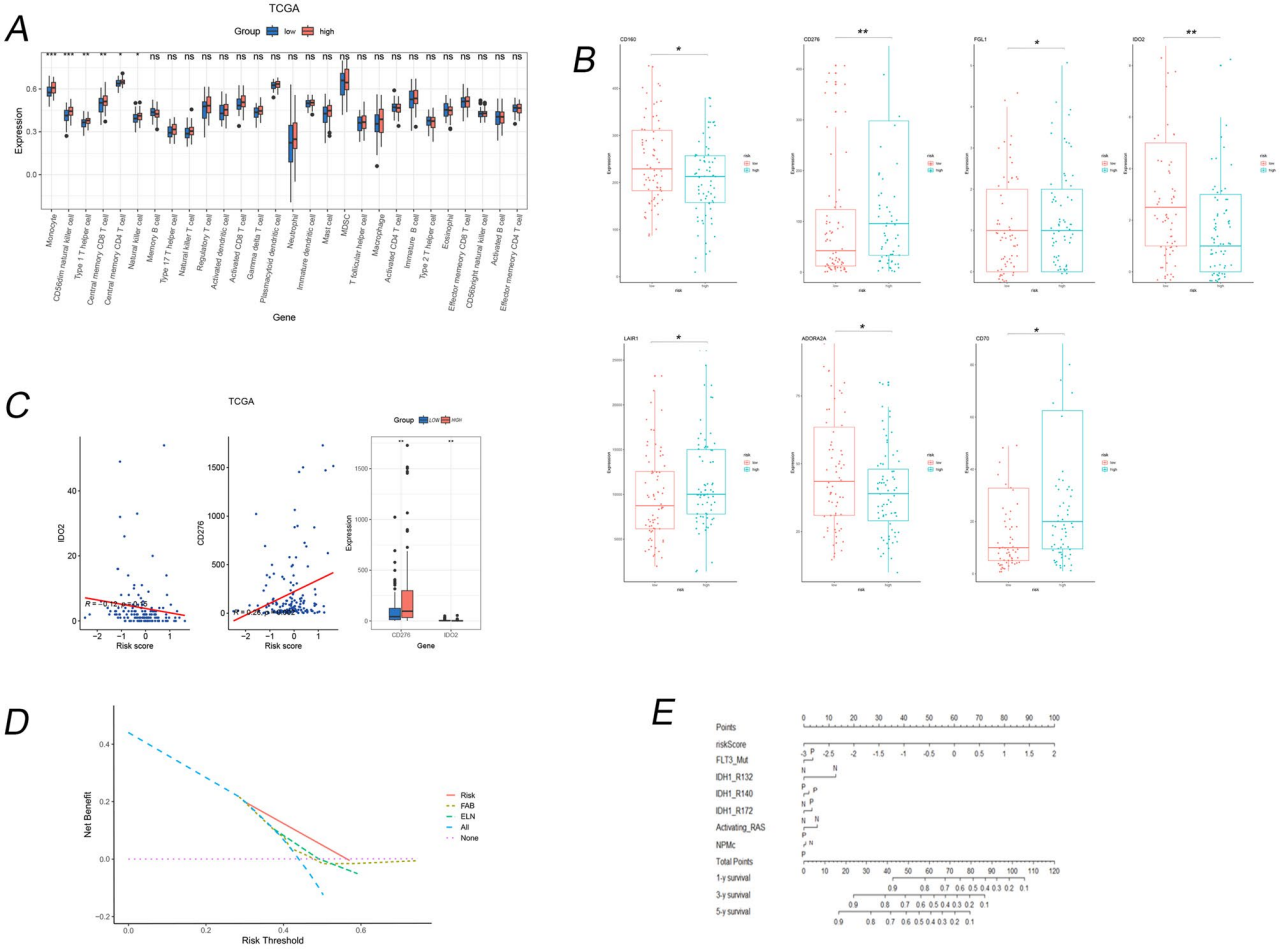
Immune characteristics of different risk score model. (A) The expression of immune cells in different risk groups. (**B**) Statistically differentially expressed immune checkpoint (IC) in different risk groups. A total of seven IC differed in expression levels between high and low risk groups. (**C**) Correlation of *IDO2* and *CD275* with risk model, expression in high and low risk groups respectively. (**D**) Decision curve analysis (DCA) plot. (**E**) The nomogram based on risk score model and other prognosis-related genes.

After analyzing the composition of immune cells in different risk groups, we found that Monocyte, CD56dim natural killer cell, Type1 T helper cell, central memory CD8, central memory CD4 T cells and natural killer (NK) cells were more abundant in the high-risk subgroup (Fig. 6A).

Next, we explore the relationship between risk group and immune checkpoint (IC) expression. As a result, the following seven ICs differentially expressed in different risk groups *LAIR1*, *CD276*, *CD70*, *CD160*, *ADORA2AM*, *IDO2*, *FGL1* (Fig. 6B). Among them, *CD276* and *IDO2* were the most statistically significant, with *CD276* highly expressed in the high-risk group and *IDO2* highly expressed in the low-risk group (*P* < 0.01, Fig. 6C).

### Nomogram and DCA of risk score model

The DCAs demonstrated that the performance of the nomogram closely resembled that of the French American British (FAB) classification or European Leukemia Net (ELN) recommendations in AML patients (Fig. 6D). Alongside the risk score, there exist multiple well-known prognostic factors for genes, including *FLT3* mut, *NPM1*, and *IDH2*. To predict 1-, 3-, and 5-year overall survival (OS), we developed a nomogram by incorporating the risk signature and the aforementioned genes. Within the nomogram, each signature was assigned points based on its risk contribution to OS (Fig. 6E).

## Discussion

AML is a hematological malignancy originating from hematopoietic stem/progenitor cells. The heterogeneous nature of AML is characterized by a wide range of clinical manifestations and prognoses. Notably, advancements in AML chemotherapy response have been made by targeting specific markers, particularly those expressed on LSCs or associated signaling pathways. Consequently, investigating leukemia markers, specifically those expressed on LSCs, is a crucial area of ongoing research. Among the recognized surface markers on LSCs, *Tim-3* has emerged as a significant player in AML progression^9,10^. Meta-analyses exploring *Tim-3* have revealed a correlation between *Tim-3* overexpression and an unfavorable prognosis across various cancer types^16^. However, further investigation is required to understand the clinical and biological characteristics of *Tim-3* specifically in the context of AML. A recent study highlighted the potential significance of upregulated *Tim-3* as a prognostic marker, indicating an unfavorable prognosis for individuals diagnosed with AML. Moreover, the biological properties of *Tim-3* were found to be associated with immune responses and signaling pathways involved in the regulation of LSCs^13^. The identification of hub genes displaying differential expression levels in relation to *Tim-3* may offer valuable opportunities to deepen our understanding of prognosis and the role of LSCs within the Tumor Immune Microenvironment (TME) of AML.

In our study, DEGs grouped by high and low *TIM-3* expression were enriched in the following 3 pathways: positive regulation of cytokine production, T cell activation, leukocyte cell–cell adhesion. We then explored the immune cells and immune microenvironment scores in the two groups with high and low *TIM-3* expression and found that they differed in both groups. Based on our conjecture that variations in the TME play a dominant role in the high and low expression groups, our focus shifted towards identifying hub genes associated with the immune microenvironment. By exploring the immune-related gene expression profiles, we aimed to pinpoint genes that potentially exert a significant influence on the TME.

TME is influenced by a multitude of genes, and the use of WGCNA provides a valuable approach for identifying potential immune-related biomarkers^17^. In our study, we employed WGCNA using TCGA datasets to identify hub genes within two immune-related modules. Subsequently, we filtered these genes based on their impact on patient overall survival (OS). By utilizing these identified genes, we developed a risk score model that incorporates their expression levels. The constructed risk score model emerged as a robust prognostic biomarker for AML. It exhibited favorable survival outcomes in the low-risk group and poorer survival outcomes in the high-risk group, as observed across both the TCGA and GEO cohorts. Moreover, the risk score model demonstrated notable efficacy in predicting survival rates at 1, 3, and 5 years, with a particular emphasis on the 5-year survival endpoint.

Following the categorization of samples into high and low-risk groups based on their respective risk scores, we proceeded to conduct pathway analysis on the DEGs within each group. Our analysis revealed significant enrichment of pathways related to leukocyte cell–cell adhesion, regulation of T cell activation, and I-κB kinase/NF-κB signaling. Notably, these pathways exhibited substantial enrichment in the high-risk group, indicating their potential involvement in disease progression and prognosis. The role of adhesion is to regulate cellular connections and their interaction with the extracellular matrix, thereby influencing the localization of cells in their respective environments. In AML, the processes of quiescence, migration, and adhesion within the bone marrow (BM) exert significant influence^18^. Adhesion molecules involved in the interaction between HSCs or LSCs and the BM microenvironment have been implicated in LSC survival and resistance to chemotherapy^19,20^. The adhesion-dependent survival of LSCs in AML patients contributes to the development of treatment resistance and reduced overall survival. Therefore, targeting the adhesion process holds potential for therapeutic interventions.

The immune microenvironment of the BM in AML patients exhibits distinct characteristics compared to that of healthy individuals. Notably, activated regulatory T cells (Treg) are present in the immune microenvironment of AML, promoting the emergence of suppressive subsets^21^. High expression of Treg in AML impacts the response to induction chemotherapy and contributes to synergistic and amplified drug resistance^22^. Primary AML CD34(+) cells exhibit detectable NF-kappaB activity^23^. Furthermore, LSCs aberrantly express NF-kappaB, rendering it a potential therapeutic target in AML^23,24^. Interestingly, the stimulation of *TIM-3* by Gal-9 has been found to co-activate signaling pathways for LSC self-renewal, specifically NF-κB and β-catenin^9^. The hub genes associated with *TIM-3* expression in our risk model suggest the involvement of the NF-κB signaling pathway in the AML microenvironment. Furthermore, we performed PPI analyses of DEGs in the high- and low-risk groups and found that FGF13 and BMP4, PPARG in up gene network, NRXN2, NLGN4Y,CNTN1, EGFR,ERBB2,ERBB3 in down gene network.

To further investigate the immunological characteristics associated with the risk groups, we conducted an analysis of gene mutations within each group. Correlation analysis of clinical parameters results showed that the risk score was correlated with patients’ gene mutation(*FLT3*, *IDH1*, *NPM1c*). Our findings revealed the presence of the *NPM1* mutation, which is known to confer a favorable prognostic effect in AML, within the high-risk group. Interestingly, we also observed the presence of the *FLT3* mutation within the high-risk group. *FLT3* mutations are the most commonly observed genetic aberrations in AML and are associated with a poor prognosis^25^. This suggests that the poor prognosis observed in the high-risk group cannot be reversed by the presence of the *NPM1* mutation^26,27^. Mutations in *TP53* were found to be more frequent in the high-risk group. AML patients with *TP53* mutations have been associated with poor outcomes, and the specific class-defining mutations have an independent and additive impact on prognosis^27^. *TP53* mutations have been incorporated into the risk stratification guidelines for AML recommended by the 2017 European Leukemia Net (ELN)^28^.

Furthermore, we observed variations in the prevalence of gene mutations between the low-risk and high-risk groups. Specifically, *IDH2* mutations were more prevalent in the low-risk group, suggesting their potential as therapeutic targets to improve overall survival in AML patients within this group. We specifically identified an *IDH2* mutation within the low-risk group. These *IDH* mutations disrupt the differentiation process in AML cells by causing abnormal epigenetic regulation^29^. Encouragingly, substances such as enasidenib, which serve as inhibitors of *IDH2*, have shown promising remission rates and have received approval from regulatory authorities in the United States^30^. *MUC16*, also known as *CA125*, exhibits distinct expression patterns under normal physiological conditions as well as during tumorigenesis. While *MUC16* plays a protective role in healthy physiology, it has been implicated in disease progression and metastasis in various cancers. Its abnormal overexpression makes it an appealing target for diagnostic purposes and immune therapy^31,32^.

TME can provide valuable insights for the development of innovative strategies to treat AML and improve the effectiveness of immunotherapies. We observed differences in the composition of immune cells between the two risk groups. Specifically, the high-risk subgroup exhibited higher levels of monocytes, CD56dim natural killer cells, Type 1T helper cells, central memory CD8 T cells, central memory CD4 T cells, and natural killer (NK) cells. These cell types are involved in the inflammatory response. Chronic inflammation has been associated with the development of premature aging phenotypes and myeloid neoplasms. Soluble cytokines such as tumor necrosis factor (TNF), interferons (IFNs), and interleukin 6 (IL6) have been linked to hematologic neoplasms related to aging, including myeloproliferative neoplasms (MPN), myelodysplastic syndromes, and AML^33–35^. In a recent study, individuals who showed positive clinical responses to immunotherapy involving αCTLA-4 and/or αPD-1 antibodies along with a hypomethylating agent (HMA) demonstrated a significant increase in the population of central memory (CM) CD8+ T cells^36^.

The advent of immune checkpoint inhibitors has revolutionized the treatment of previously untreatable malignancies in cancer immunotherapy. In this study, we aimed to explore the relationship between established immunotherapy prediction biomarkers and the risk group classification. Our findings revealed a significant correlation between the risk group and two immune checkpoints: *IDO2* and *CD276*. *CD276*, also known as *B7-H3*, belongs to the *B7* family of immune checkpoint molecules. High expression of *B7-H3* was found to be associated with poor overall survival in AML patients^37^. Our study supports these observations by showing a positive association between *CD276* expression and the risk score. Additionally, a significant proportion of AML patients exhibit elevated levels of *B7-H3* on leukemic blasts, making it a promising target antigen for the development of CAR-T therapy specifically directed towards AML^38^. Evidence suggests that *IDO2* exhibits lower efficiency in metabolizing l-tryptophan compared to *IDO1*. Instead, its activities are believed to be influenced by interactions with other yet unidentified proteins, which may vary in different inflammatory and neoplastic contexts. Consequently, identifying the interactome and function of *IDO2* in different neoplastic conditions may pave the way for the development of novel treatment approaches^39,40^. *CD276* and *IDO2* exhibit promise as viable targets for immunotherapeutic interventions in AML.

Minimal residual disease (MRD) should indeed be taken into account as a prognostic index. Unfortunately, however, MDR was not found in the corresponding clinical features of the TCGA and several other GEO datasets. However, comparing the blasts of PB and BM in the high- and low-risk groups suggested that their high values were independently associated with the high- and low-groups. Blast of PB and BM may have less effect on high and low risk. Besides, Multivariate survival analysis found that risk prognostic model was the independent prognostic factor for AML patients. To further understand the predictive ability of the model for prognosis, we compare it with the two standard scenarios of ELN recommendations and FAB classification in DCA curves. We can easily find that the risk prognosis model is closer to the curves of these two scenarios, which demonstrates that the risk score model has a better ability to predict prognosis. In addition to the risk score, there are numerous known prognostic factors for genes, so we selected the genes in ELN recommendations for the nomogram construction. This nomogram combined with genes based on ELN recommendations can better predict the prognosis of AML patients.

Risk score group based on the level of *TIM-3* expression is a promising immune-related prognostic model. We found that the high-risk group is involved in the process of cell adhesion and the process of inflammatory response, such as migration, homing, and quiescence of LSCs, which plays an important role in AML. The results implied that high-risk group was characteristics of active tumor progression. The two immune checkpoints: *IDO2* and *B7-H3* would be the potential immune therapy targets. The risk score model demonstrated superior predictive power in AML with high *TIM-3* expression. The utilization of a risk score model exhibits potential in distinguishing the immune and molecular characteristics of patients, thereby enabling the prediction of patient outcomes. Further studies are needed to clarify this assertion.

## Methods

### Patients and datasets

In order to obtain clinical insights pertaining to AML, a cohort consisting of 140 cases was examined through the utilization of RNA sequencing (RNA-seq) data and gene mutation information. The database of choice for this investigation was the Cancer Genome Atlas (TCGA). Furthermore, to fully validate the reliability of the model, we chose different Gene Expression Omnibus (GEO) databases as external validation (GSE71014, GSE12417, GSE146173). This included samples of cytogenetically normal AML (CN-AML) and secondary AML, treatment-resistant AML, with a view to approximating real-world.

### Functional enrichment analysis

To identify a collection of differentially expressed genes (DEGs), a rigorous differential expression analysis was performed. Subsequently, the Gene Ontology (GO) and Kyoto Encyclopedia of Genes and Genomes (KEGG) analyses^41^, widely recognized methodologies in the field, were conducted. The clusterProfiler package, implemented in the R programming language, served as the tool of choice for these analyses.

### Protein–Protein interaction networks

We added Protein–Protein Interaction Networks analysis for DEGs in high and low risk groups through STRING websites.

### Evaluation of immune cell fractions

Immunosuppression within the microenvironment of AML is thought to involve a range of cell types, including myeloid-derived suppressor cells (MDSCs), regulatory T cells (Tregs), natural killer (NK) cells, neutrophils, and various other cells. To investigate the composition of immune cells mentioned above, an analytical approach known as single-sample gene set enrichment analysis (ssGSEA) was employed. This approach utilizes the R package "GSVA" to quantify the extent of immune cell enrichment within the microenvironment, based on gene expression levels obtained from a single tumor sample^42^. Furthermore, the R package "estimate" was utilized to evaluate the ImmuneScore, a measure indicative of immune cell infiltration within the tumor microenvironment.

### Identification of hub genes

By employing RNA-seq data obtained from AML samples sourced from the TCGA database, we conducted an investigation to identify differentially expressed genes based on the categorization of TIM-3 expression levels into “High” and “Low” groups. The identification of such genes was accomplished using the R package “DESeq” with statistical significance defined as *P* < 0.05 and |log2FC|> 2 ^43^.

To explore the relationship between gene expression patterns and Gene Ontology (GO) findings, we employed WGCNA^17^. This analysis focused on identifying hub genes within immune cells and the microenvironment. Initially, a similarity matrix was computed, which was subsequently transformed into an adjacency matrix. To facilitate this transformation, a signed network type was utilized along with a soft threshold of 5. The resulting adjacency matrix was further transformed into a topological matrix utilizing the topological overlap measure (TOM) to quantify the degree of gene linkage. Modules were identified through the implementation of a dynamic pruning tree, which grouped genes based on the 1-TOM distance. Through this process, a total of 11 modules were identified by adjusting the merging threshold function to 0.25.

Following the completion of the WGCNA analysis, we focused on genes belonging to modules that exhibited significant associations with ImmuneScore and ESTIMATEScore. Specifically, our attention was directed towards the "blue" and "turquoise" modules. To construct a network, edges with a weight greater than 0.4 were considered, taking into account the genes from these significantly related modules. Subsequently, we narrowed our analysis to hub genes within these modules that demonstrated significant Kaplan–Meier (K–M) survival analysis results. This rigorous selection process resulted in the identification of 16 immune-related hub genes that displayed significant associations with survival (*P* < 0.05, log-rank test) and were thus chosen for further analysis.

### Construction and validation of the prognostic risk-scoring model

In order to identify hub genes that significantly influenced overall survival (OS), we employed multivariate Cox regression analysis. These hub genes were then utilized to construct a model. To generate the most optimal model, we utilized the R package "My.stepwise" for screening hub genes associated with OS. The resulting model was defined by the following formula: (Riskscore = ∑^N^_i=1_ (NCoef_i_, × Exp_i_,)). To evaluate the predictive capability of the model, Kaplan–Meier survival curves and log-rank tests were performed on both the TCGA and GEO cohorts. Furthermore, time-dependent ROC curve analyses were conducted using the R package "timeROC" to calculate the area under the curve (AUC) and compare the prognostic value among different risk groups.

### Comprehensive analysis of molecular and immune characteristics in different risk groups

To gain insights into the gene expression profiles of samples with high (n = 71) and low (n = 70) risk scores, a differential expression analysis was initially conducted on all genes. This analysis utilized the R package “DESeq”. Subsequently, gene set enrichment analysis (GSEA) was performed using the R package “clusterProfiler” and the KEGG database to identify signaling pathways associated with the differentially expressed genes. Significance was defined as *P* < 0.05 and FDR < 0.25. Additionally, to delve further into representative gene sets, we conducted single-sample gene set enrichment analysis (ssGSEA) using the R package “GSVA”. This analysis provided a deeper understanding of the functional enrichment of the identified gene sets.

To investigate the differences in immune cell composition between the two risk groups, we conducted a comparative analysis of the relative proportions of 22 types of immune cells. This analysis aimed to gain further insights into the immune and molecular functions associated with these risk groups. Additionally, we utilized the R package “Maftools” to analyze gene mutations between the two groups, providing a comprehensive understanding of the genetic landscape.

In order to assess the prognostic value of the risk group in patients undergoing immunotherapy, we generated a correlation heatmap between immune checkpoint (IC) and risk group samples. This analysis allowed us to evaluate the relationship between the risk group and the expression of immune checkpoint genes, shedding light on potential therapeutic implications.

### Development and assessment of the nomogram

To develop a personalized prediction model for clinical events, we employed a nomogram. A nomogram is a visual representation that utilizes statistical prediction models to provide a straightforward graphical representation of the probability of specific clinical outcomes. In our study, the creation of the nomogram involved the utilization of R packages such as “survival” and “rms”.

To assess the accuracy of the nomogram in predicting survival rates (1, 3, and 5-year) for patients with AML, we conducted decision curve analysis (DCA). This analysis compared the decision curve of the nomogram with those derived from other prognostic factors, enabling an evaluation of its performance and clinical utility.

### Statistical analysis

To compare continuous variables between the two groups, an independent t-test was performed. The chi-square test was used for categorical data, where either predicted frequency was less than 5, using Fisher’s exact test. For univariate survival analysis, the Kaplan–Meier (K–M) method was employed. This method enabled the estimation of survival probabilities over time, and the log-rank test was used to assess the significance of differences in survival curves between the two groups. Cox regression was used for univariate and multivariate survival analyses. *P*-values were corrected using the Bonferroni correction method. The significance level for all statistical tests was set at a two-sided *P*-value of less than 0.05, indicating statistical significance.

### Ethics approval

All data in the database passed ethical review and informed consent before being uploaded to TCGA. Ethics Approval is allowed by Ethics Committee of Shanghai Pudong Hospital. Our research is based on open-source data and is therefore free of ethical issues and other conflicts of interest.

### Supplementary Information

Supplementary Information.

## Data Availability

In our study, TCGA (https://portal.gdc.cancer.gov) and GEO (https://www.ncbi.nlm.nih.gov/geo/.) belong to public databases. The samples involved in the database have received ethical approval. Users are free to download relevant data for research and publication of relevant articles. All data in the database passed ethical review and informed consent before being uploaded to TCGA.

## References

[1] Vetrie D, Helgason GV, Copland M (2020). The leukaemia stem cell: Similarities, differences and clinical prospects in CML and AML. Nat. Rev. Cancer.

[2] Estey EH (2018). Acute myeloid leukemia: 2019 update on risk-stratification and management. Am. J. Hematol..

[3] Jones CL (2019). Inhibition of amino acid metabolism selectively targets human leukemia stem cells. Cancer Cell.

[4] Ho TC (2016). Evolution of acute myelogenous leukemia stem cell properties after treatment and progression. Blood.

[5] Marchand T, Pinho S (2021). Leukemic stem cells: From leukemic niche biology to treatment opportunities. Front. Immunol..

[6] Zhu C (2005). The Tim-3 ligand galectin-9 negatively regulates T helper type 1 immunity. Nat. Immunol..

[7] Sakuishi K (2010). Targeting Tim-3 and PD-1 pathways to reverse T cell exhaustion and restore anti-tumor immunity. J. Exp. Med..

[8] Pagliano O (2022). Tim-3 mediates T cell trogocytosis to limit antitumor immunity. J. Clin. Investig..

[9] Kikushige Y (2015). A TIM-3/Gal-9 autocrine stimulatory loop drives self-renewal of human myeloid leukemia stem cells and leukemic progression. Cell Stem Cell.

[10] Kikushige Y (2010). TIM-3 is a promising target to selectively kill acute myeloid leukemia stem cells. Cell Stem Cell.

[11] Jan M (2012). Clonal evolution of preleukemic hematopoietic stem cells precedes human acute myeloid leukemia. Sci. Transl. Med..

[12] Haubner S (2019). Coexpression profile of leukemic stem cell markers for combinatorial targeted therapy in AML. Leukemia.

[13] Wu Z (2023). Upregulation of Tim-3 is associated with poor prognosis in acute myeloid leukemia. Cancer Med..

[14] Wang Z, Chen J, Wang M, Zhang L, Yu L (2021). One stone, two birds: The roles of Tim-3 in acute myeloid leukemia. Front. Immunol..

[15] Prada-Arismendy J, Arroyave JC, Rothlisberger S (2017). Molecular biomarkers in acute myeloid leukemia. Blood Rev..

[16] Zang K (2021). TIM-3 as a prognostic marker and a potential immunotherapy target in human malignant tumors: A meta-analysis and bioinformatics validation. Front. Oncol..

[17] Langfelder P, Horvath S (2008). WGCNA: an R package for weighted correlation network analysis. BMC Bioinform..

[18] Gruszka AM, Valli D, Restelli C, Alcalay M (2019). Adhesion deregulation in acute myeloid leukaemia. Cells.

[19] Ashok D, Polcik L, Dannewitz Prosseda S, Hartmann TN (2021). Insights into bone marrow niche stability: An adhesion and metabolism route. Front. Cell Dev. Biol..

[20] Grenier JMP, Testut C, Fauriat C, Mancini SJC, Aurrand-Lions M (2021). Adhesion molecules involved in stem cell niche retention during normal haematopoiesis and in acute myeloid leukaemia. Front. Immunol..

[21] Guo R (2021). Single-cell map of diverse immune phenotypes in the acute myeloid leukemia microenvironment. Biomark. Res..

[22] Szczepanski MJ (2009). Increased frequency and suppression by regulatory T cells in patients with acute myelogenous leukemia. Clin. Cancer Res..

[23] Guzman ML (2001). Nuclear factor-kappaB is constitutively activated in primitive human acute myelogenous leukemia cells. Blood.

[24] Jin Y (2010). Antineoplastic mechanisms of niclosamide in acute myelogenous leukemia stem cells: Inactivation of the NF-kappaB pathway and generation of reactive oxygen species. Cancer Res..

[25] Zhao JC (2022). A review of FLT3 inhibitors in acute myeloid leukemia. Blood Rev..

[26] Dohner K (2005). Mutant nucleophosmin (NPM1) predicts favorable prognosis in younger adults with acute myeloid leukemia and normal cytogenetics: Interaction with other gene mutations. Blood.

[27] Papaemmanuil E (2016). Genomic classification and prognosis in acute myeloid leukemia. N. Engl. J. Med..

[28] Dohner H (2022). Diagnosis and management of AML in adults: 2022 recommendations from an international expert panel on behalf of the ELN. Blood.

[29] McMurry H, Fletcher L, Traer E (2021). IDH Inhibitors in AML-promise and pitfalls. Curr. Hematol. Malig. Rep..

[30] Bose P, Vachhani P, Cortes JE (2017). Treatment of relapsed/refractory acute myeloid leukemia. Curr. Treat. Options Oncol..

[31] Felder M (2014). MUC16 (CA125): Tumor biomarker to cancer therapy, a work in progress. Mol. Cancer.

[32] Aithal A (2018). MUC16 as a novel target for cancer therapy. Expert Opin. Ther. Targets.

[33] Puissant A, Medyouf H (2022). Walking the tightrope: Balancing delicate inflammation response to eradicate acute myeloid leukemia. Cancer Discov..

[34] Wu J (2020). A single-cell survey of cellular hierarchy in acute myeloid leukemia. J. Hematol. Oncol..

[35] Mueller SN, Gebhardt T, Carbone FR, Heath WR (2013). Memory T cell subsets, migration patterns, and tissue residence. Annu. Rev. Immunol..

[36] Lee SE (2023). Immunologic predictors for clinical responses during immune checkpoint blockade in patients with myelodysplastic syndromes. Clin. Cancer Res..

[37] Wang P (2023). Optimal combination of immune checkpoint and senescence molecule predicts adverse outcomes in patients with acute myeloid leukemia. Ann. Med..

[38] Lichtman EI (2021). Preclinical evaluation of B7–H3-specific chimeric antigen receptor T cells for the treatment of acute myeloid leukemia. Clin. Cancer Res..

[39] Mondanelli G (2021). Current challenges for IDO2 as target in cancer immunotherapy. Front. Immunol..

[40] Platten M, Nollen EAA, Rohrig UF, Fallarino F, Opitz CA (2019). Tryptophan metabolism as a common therapeutic target in cancer, neurodegeneration and beyond. Nat. Rev. Drug Discov..

[41] Yu G, Wang LG, Han Y, He QY (2012). clusterProfiler: An R package for comparing biological themes among gene clusters. OMICS.

[42] Hanzelmann S, Castelo R, Guinney J (2013). GSVA: Gene set variation analysis for microarray and RNA-seq data. BMC Bioinform..

[43] Anders S, Huber W (2010). Differential expression analysis for sequence count data. Genome Biol..

