# Supplementary material for: Identification and validation of a prognostic risk-scoring model based on the level of TIM-3 expression in acute myeloid leukemia
Source: Sci Rep. 2023 Sep 20;13:15658. doi: 10.1038/s41598-023-42700-2 (PMC10511414; doi:10.1038/s41598-023-42700-2)
Supplement: Supplementary file 1 — Supplementary Information. [file 41598_2023_42700_MOESM1_ESM.docx]

**Supplementary Table1**

| Gene Name | coefficient |
| --- | --- |
| TET2 | -0.0001643187 |
| MIOS | 0.0031712272 |
| F3 | 0.0010013229 |
| EXOC6B | -0.0025028036 |
| FAM215A | 0.0336304105 |
| B3GALT4 | 0.0101334988 |
| ZMAT5 | -0.0079054463 |
| DAND5 | 0.0237360206 |
| ANAPC7 | -0.0009175948 |
| SNX30 | -0.0002753708 |
| CORO2B | 0.0016750071 |
| EFCAB12 | 0.0050340658 |
| ATP6V1G1 | 0.0005406299 |
| PAK4 | 0.0013652052 |
| MAN2B2 | -0.0003060998 |
| KCNJ6 | -0.0008459643 |

Supplementary Table1. The coefficient of risk score model*. The coefficient for each gene is shown in the table. The prognostic model is shown below.

*Model = expression level of TET2*(−0.0001643187) + expression level of MIOS *0.0031712272 + expression level of F3*0.0010013229 + expression level of EXOC6B*(-0.0025028036) + expression level of FAM215A*0.0336304105 + expression level of B3GALT4*0.0101334988 + expression level of ZMAT5*(-0.0079054463) + expression level of DAND5*(0.0237360206) + expression level of ANAPC7*(−0.0009175948) + expression level of SNX30*(−0.0002753708) + expression level of CORO2B*0.0016750071 + expression level of EFCAB12*0.0050340658 + expression level of ATP6V1G1*0.0005406299 + expression level of PAK4*0.0013652052 + expression level of MAN2B2*(−0.0003060998) + expression level of KCNJ6*(−0.0008459643)

**Supplementary Figure1**


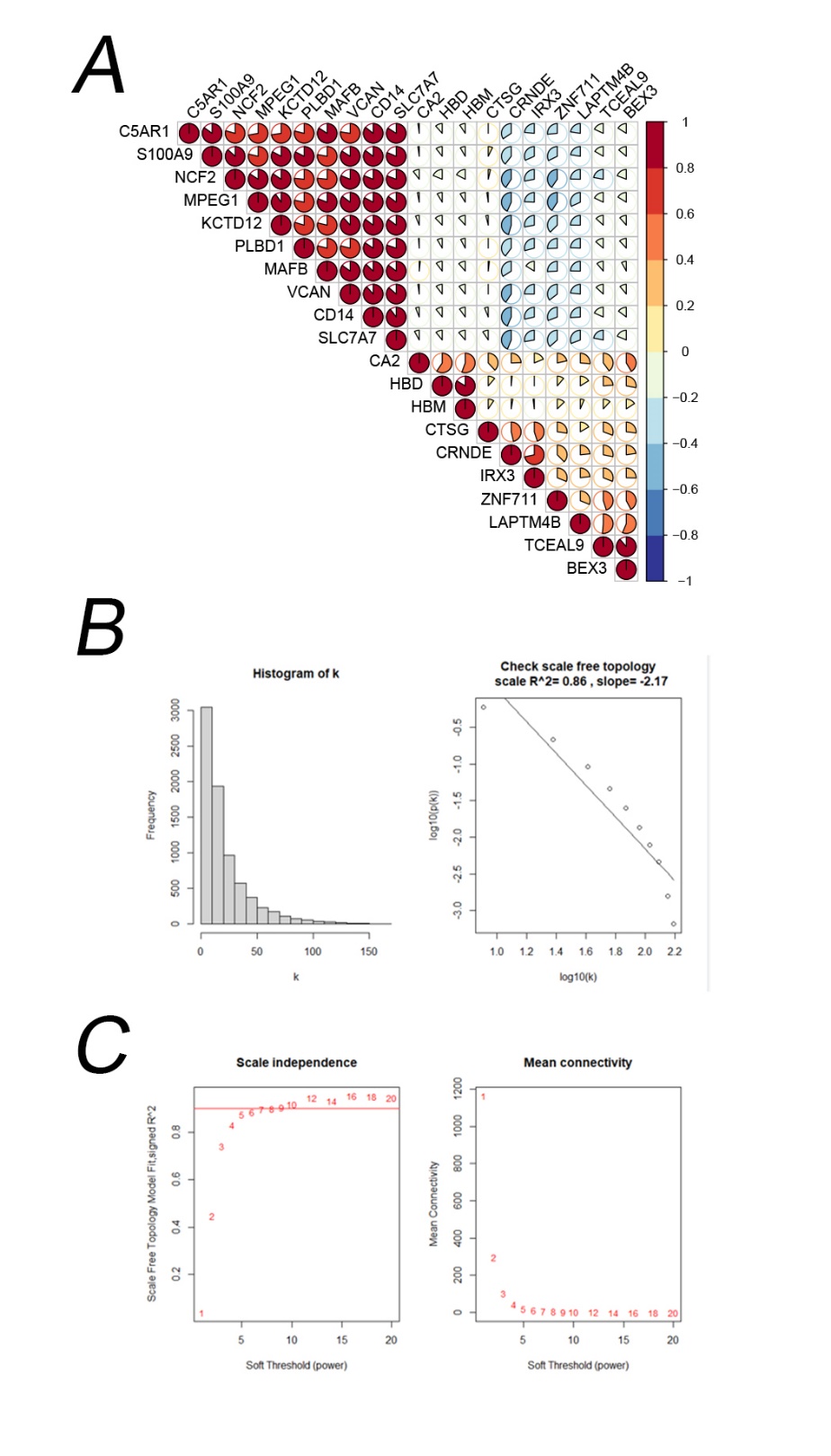


Supplementary Figure1. Correlation of DEGs in the TIM-3 expression group and related parameters of weighted gene co-expression network analysis (WGCNA). A. The correlation of top10 DEGs in High and low TIM-3 expression B. Histogram and check scale free topology C. Soft threshold and mean connectivity
